# Computational modeling of the electric potential in biological membrane. A comparison between healthy and cancerous neurons

**DOI:** 10.1186/1471-2202-12-S1-P47

**Published:** 2011-07-18

**Authors:** Thiago M Pinto, Roseli S Wedemann, Célia M Cortez

**Affiliations:** 1 Instituto de Matemática e Estatística, Universidade do Estado do Rio de Janeiro, Rio de Janeiro, RJ, 20550-900, Brazil

## 

We have studied the behavior of the electric potential profile across the membrane of the ganglion neuron and the neuroblastoma cell. We considered the physicochemical conditions during the resting and action potential (AP) states of the neuronal cells, and analyzed the influence of the fixed charges of the membrane on the surface electric potential of these cells, based on values of electric parameters obtained from experimental results. The ganglion neuron portrays a healthy neuron and the neuroblastoma cell, which is a tumorous cell, represents a pathologic neuron. We solved the non-linear Poisson-Boltzmann equation, by considering the volumetric charge densities due to charges dissolved in an electrolytic solution, and also charges fixed on both glycocalyx and cytoplasmic proteins.

Data obtained from experimental results [1,2] have been used to solve the first order ordinary differential Poisson-Boltzmann equations [3,4], which have been obtained for the regions of the membrane model we have adopted. Therefore, we have examined the influence of the electric parameters during resting and AP states, analyzing the differences between the healthy ganglion neuron and a neuroblastoma cell.

We implemented an algorithm for finding roots of functions with a program in C language, to calculate the surface potentials between the glycocalyx and the bilayer, and between the bilayer and the cytoplasm, regions of the membrane. The surface potential between the electrolytic region and the glycocalyx has been calculated from electrophoretic experimental data. We have calculated the potential profiles using the Runge-Kutta method, also implemented in C language.

We analyzed the electric potential profile across the membranes. Comparing both neuronal types, we verified that the gradual potential fall from the electrolytic region to the surface of glycocalyx is higher for the ganglion neuron than for the neuroblastoma cell, and both curve shapes are similar. Through the glycocalyx we could see that the fall continues for the ganglion neuron, but this fall is negligible for the cancerous cell. The intracellular potential, during the resting state, increases exponentially from the bilayer surface to the bulk cytoplasmic region.

During the resting state, the net value of the protein charge in the cytoplasm is predominantly negative [4]. However, in our neuron model the contribution of these charges to the inner potential profile was smaller than the effect of the fixed charges in the inner surface of the bilayer, due to the curvature of the potential in this region, whereas the calculated potential on the surface between the bilayer and the cytoplasm was smaller than the potential in the bulk region. It is known that the neuroblastoma cells, like all the other cancerous cells, multiply themselves quickly. Alterations of the dynamics of the cellular multiplication cause changes in the synthesis, structure and promotes degradation of the membrane components [5], which results in deformations in structure and composition of the plasma membrane.

## Conclusions

Our results may also contribute to the understanding of the neuroblastoma resistance to certain chemotherapeutic treatments. The small change of the surface potential as a response to changes in the culture (pH, for instance) and in the fixed electric charges, due to alterations in the composition and structure of the membrane, may be the electric property responsible for the low pharmacological response.
